# CXCL8 – The First Chemokine

**DOI:** 10.3389/fimmu.2015.00285

**Published:** 2015-06-08

**Authors:** Marco Baggiolini

**Affiliations:** ^1^Theodor Kocher Institute, University of Bern, Bern, Switzerland

**Keywords:** CXCL8, chemokines, neutrophil leukocytes, Naf, IL8

After spending 2 years at the Rockefeller University in New York, as a research associate in the laboratory of Christian de Duve, I accepted an offer from Sandoz Ltd., which was attractive in terms of space, equipment, and research facilities, and returned to Switzerland. *Beatrice Dewald*, joined my laboratory after several years at NYU and Vanderbilt University, and we continued our studies of the enzymes of neutrophil granulocytes initiated at Rockefeller, with particular attention to neutral proteinases, their release and their role in tissue damage and inflammation. We went back to academia in 1983, when I became director of the Theodor Kocher Institute, a unique institution for graduate studies, associated with the Faculties of Sciences and Medicine of the University of Bern, which everybody called TKI. Theodor Kocher, both surgeon and scientist, was awarded the Nobel Prize in 1909 “*for his work on the physiology, pathology, and surgery of the thyroid gland*.” He firmly believed in the role of basic research for medical progress, and donated his prize money as an initial contribution to the construction of a dedicated interfaculty institute.

At the TKI, research on human leukocytes in inflammation and host defense continued with new colleagues, *Alfred Walz*, head of a laboratory for biochemistry and molecular biology, working on interferon and cytokines, and two physicists, *Dave Deranleau* and *Vinzenz von Tscharner*, who developed methods and instruments for the real-time analysis of cell activation. My experience in the pharmaceutical industry was an asset, but the TKI had something more to offer: outstanding Ph.D. students and postdoctoral fellows, a major resource for innovation. The young associates and the students kept me close to the bench and open to lateral thinking.

I am expected to narrate how the first chemotactic protein was discovered. In Bern, it all began with a surprising encounter. One evening, on the stairs of the TKI, I bumped into *Paul Imboden*, a Ph.D. student in the laboratory of *Alfred Walz*, who told me he had found in human monocyte cultures an agent that stimulates neutrophil leukocytes. No real surprise, there, I thought. Still, I proposed to test on neutrophil leukocytes the effects of the new substance and of chemotactic agonists that were known, i.e., C5a, fMet–Leu–Phe, platelet-activating factor, and leukotriene B_4_. The new substance was a protein that triggered responses similar to those induced by common chemotactic agonists, but acted through a yet unknown G-protein-coupled receptor. Unlike the common agonists, which induce migration of different granulocytes and even of monocytes, the novel protein was specific for neutrophils, and we thus called it NAF, for “neutrophil-activating factor.” The observed selectivity for a single type of white cells was an important new finding. We imagined, with some optimism, that NAF could be a prototype for a novel class of chemotactic proteins, and thought that the search for proteins related to NAF was going to pay off. Analogs were indeed identified, and the laboratories that pioneered such progress agreed to name the new proteins “chemokines,” in abbreviation of “chemotactic cytokines.”

The characterization of NAF had to be completed first. *Alfred Walz* prepared a highly purified sample of the protein, and I arranged for micro-sequencing at the Sandoz Research Institute in Vienna. To our surprise, we were not alone! Between December 1987 and April 1988, the newly discovered protein was presented in four independent papers (1–4). It was an unusual, choral announcement of four matching partial amino-acid sequences. Furthermore, the sequence data were in agreement with the cDNA-deduced sequence of a secretory protein of 99 residues that had been published a few months before. The protein was homologous to β-thromboglobulin, but its properties and function were not identified (5).

**Figure 1:**
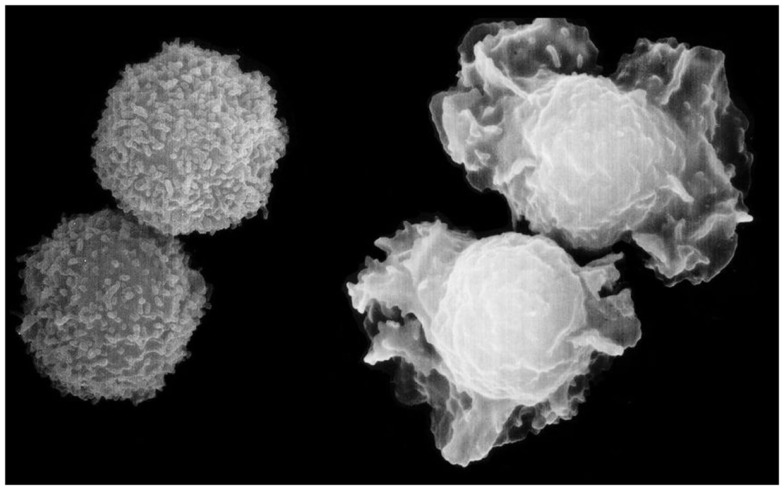
**Shape change of neutrophils, the most elegant response to chemo-attractants, observed within seconds of challenge, and characterized by the protrusion and retraction of ectoplasmic extensions, due to polymerization and breakdown of actin**. The protrusions appear to function like a swimmer’s arms and legs. Turbidity recordings suggest that the changes are synchronized, possibly to facilitate migration (11). Scanning electron micrograph by *Matthias Wyman*, a former Ph.D. student at TKI, now professor at the University of Basel.

*Ivan Lindley*, *Heinz Aschauer*, and other colleagues at the Sandoz Research Institute in Vienna, who had sequenced our purified protein, went a few steps further. They synthesized a gene coding for the 72-residue NAF, which they cloned and expressed. The recombinant protein was analyzed at the TKI, and found to be identical in activity and potency to purified, natural NAF (6).

The first chemokine had been thoroughly characterized, but still did not have a name. None of the acronyms used in the papers reporting isolation, sequencing, cloning, and expression (MDNCF, NAF, MONAP, LYNAP, etc.) being suitable, the new protein ended up with a fancy but misleading name: “interleukin 8.” It was the first and the last chemokine to be taken for an interleukin.

Four papers putting forward the same message indicated that the chemokine area was important and competitive. I went to Frederick to see *Ed Leonard*, *Jo Oppenheim*, and colleagues and discuss possible collaborations. For me, that visit was also a chance to meet *Teizo Yoshimura* and *Kouji Matsushima*. We decided to continue our friendly relations without a formal collaboration, which was reasonable since there was no way of knowing how things would develop in the field and how research in our laboratories would evolve. In line with TKI traditions, we characterized NAF/IL8 using biochemical and biophysical methods, as shown in the comparison of neutrophil responses to NAF/IL8 and fMet–Leu–Phe by Thelen et al. (7). Real-time recordings of changes in cell shape, cytosolic free calcium levels, superoxide formation, and granule enzyme release showed that the responses to both chemo-attractants followed similar kinetics. In addition, the effects of both agonists were inhibited to a similar extent by pre-treating the neutrophils with *B. pertussis* toxin and other inhibitors of signal transduction. Despite the similarities in response quality, we observed a clear difference between the two agonists in terms of potency, with NAF/IL8 being 10–30 times more effective than fMet–Leu–Phe.

I emphasized that NAF/IL8 is highly selective for neutrophils, but I cannot end this brief account without pointing out that the first chemokine had, in fact, additional attractant properties, with unique scientific implications: for one thing, it attracted two brilliant scientists from Vancouver to Bern, *Bernhard Moser* and *Ian Clark-Lewis*. They were both primarily interested in NAF/IL8 and wanted to identify its receptor. They also expected to find new chemokines and new receptors, and eventually to study the structural determinants for receptor recognition and activation. *Bernhard Moser* went to school in Bern, studied at ETH Zurich, and obtained a PhD degree at the University of British Columbia before returning to Bern to clone and characterize chemokine receptors, and to study white cell traffic in immune defense. *Ian Clark-Lewis* was introduced to immunology at the famous Walter and Eliza Hall Institute of Medical Research in Melbourne and specialized in chemical protein synthesis in the US and in Canada. At the Biomedical Research Centre of the University of British Columbia, he established an impressive facility for solid-phase protein synthesis. Chemokines impressed him as a promising area for studying structure-activity relations and for the design of chemically-modified analogs including receptor antagonists.

From among the thirty and more publications witnessing the productive collaboration between *Ian*, *Bernhard* and other scientists at the TKI for more than a decade, I shall quote three highlights relating to NAF/IL8. Early structure-activity relation studies identified the short amino-terminal sequence preceding the first cysteine as the site for receptor binding and triggering (8), a principle that turned out to be valid for the whole chemokine family, underscoring the prototypical value of NAF/IL8. A very extensive study using a large sample of synthetic analogs with single amino-acid exchanges revealed that except for the cysteines and the ELR motif no other residue appeared to be required for NAF/IL8 receptor binding and activity (9). The same study showed, in addition, that IP10 (a CXC chemokine that does not activate neutrophils) can be modified to a potent attractant of neutrophils by insertion of discrete sequence domains taken from the NAF/IL8 amino-terminal loop (9). *Ian* also answered a fundamental question that was raised after observing that NAF/IL8 forms dimers in solution: do chemokines act as monomers or dimers? By replacing Leu in position 25 with N-methyl-Leu in the NAF/IL8 sequence, he created a derivative that could not dimerize but nevertheless retained full activity (10), indicating that NAF/IL8 binds to its receptors and trigger responses as a monomer. For *Bernhard Moser* the work on IL8 and receptors was a sort of high-level warming up. Bernhard’s major achievements came a few years later, with *Marcel Loetscher* and *Pius Loetscher* as associates, and several Ph.D. students, after moving on to arenas, which were increasingly populated by lymphocytes.

*Ian Clark-Lewis* died prematurely in 2002. He is much missed by those who worked with him, and saw him as a distinguished TKI-member from the West coast. He did not mind long-distance travel, and visited us regularly. He frequently took time to discuss scientific issues on the phone, in long, remarkable conversations. From Vancouver, he had set up a productive network of contacts and friendly relations with many of us.

## Conflict of Interest Statement

The author declares that the research was conducted in the absence of any commercial or financial relationships that could be construed as a potential conflict of interest.
